# Characterization of the IncX3 Plasmid Producing *bla*_NDM–7_ From *Klebsiella pneumoniae* ST34

**DOI:** 10.3389/fmicb.2020.01885

**Published:** 2020-08-05

**Authors:** Qiong Chen, Jiawei Zhou, Shenghai Wu, Yunxing Yang, Daojun Yu, Xianjun Wang, Min Wu

**Affiliations:** ^1^Department of Laboratory Medicine, Affiliated Hangzhou First People’s Hospital, Zhejiang University School of Medicine, Hangzhou, China; ^2^State Key Laboratory for Diagnosis and Treatment of Infectious Diseases, The First Affiliated Hospital, College of Medicine, Zhejiang University, Hangzhou, China; ^3^Department of Infectious Diseases, Affiliated Hangzhou First People’s Hospital, Zhejiang University School of Medicine, Hangzhou, China

**Keywords:** metallo-β-lactamase, NDM-7, IncX3, carbapenemase-producing *Klebsiella pneumoniae*, ceftazidime-avibactam

## Abstract

Carbapenemase-producing *Klebsiella pneumoniae* has been a major clinical threat worldwide because therapeutic options are limited. Although New Delhi metallo-β-lactamase (NDM) is an important carbapenemase responsible for carbapenem resistance, it is uncommon in carbapenemase-producing *K. pneumoniae* in China. In this study, we described strain HZW25, an NDM-7-producing *K. pneumoniae* strain belonging to sequence type 34 (ST34). HZW25 exhibited resistance to all β-lactams tested but was susceptible to aminoglycosides and fluoroquinolones. The whole genome of HZW25 was sequenced with Pacific Biosciences RSII SMRT technology. HZW25 was composed of one chromosomal DNA and four plasmids, and the resistance genes of HZW25 were all located on the chromosome, except *bla*_NDM–7_ was located on a conjugative plasmid belonging to type IncX3 designated P4. The results of conjugation and transformation experiments showed that *bla*_NDM–7_ could be horizontally transferred successfully from the donor strain, HZW25, to the recipient strains, *E. coli J53* and *E. coli DH5*α. The NDM variant transposable elements of the *bla*_NDM–7_-harboring plasmid P4 were the *ISL3* and *IS3000* families. The upstream region of *bla*_NDM–7_ contained Δ*ISAba125*, which was inserted near the *IS5* or Δ*IS5* sequence. Our study is the first report of metallo-β-lactamase NDM-7 in a carbapenemase-producing *K. pneumoniae* strain with ST34 in China. The emergence of NDM-producing *K. pneumoniae* would be troublesome during treatment using ceftazidime-avibactam. Therefore, the rapid and accurate identification of carbapenemase-producing *K. pneumoniae* is necessary.

## Introduction

Clinical treatment of carbapenem-resistant *Enterobacteriaceae* (CRE) is a critical challenge (Lee et al., 2016). Carbapenemase, which can be found as serine proteases and metalloproteinases, is responsible for carbapenem resistance. New Delhi metallo-β-lactamase (NDM) is a metallo-β-lactamase able to hydrolyze carbapenem (Khan et al., 2017). Although NDM-producing CRE has increased globally recently, the worldwide distribution and prevalence of NDM-positive strains appear to be variable between different countries and regions (Findlay et al., 2017; Khan et al., 2017; Lazaro-Perona et al., 2017; Wang et al., 2018; Perez-Vazquez et al., 2019; Wu et al., 2019). NDM-producing CRE strains have mainly spread in South Asia, the Baltans, North Africa and the Middle East (Dortet et al., 2014; Wu et al., 2019). Chinese national surveillance of carbapenem-resistant CRE in China has shown that NDM-producing CRE are less common than KPC-producing CRE, and NDM-positive strains are mainly *E. coli* (Zhang et al., 2017, 2018; Wang et al., 2018).

Since the first report of NDM in 2009, many variant NDMs have emerged (Yong et al., 2009; Dortet et al., 2014; Khan et al., 2017; Wu et al., 2019). Compared with NDM-1, NDM-7 has only two different amino acids, including Asp130Asn and Met154Leu, and NDM-7 has more enzymatic hydrolysis activity against carbapenem (Cuzon et al., 2013; Rahman et al., 2014). NDM-7 mainly exists in the IncX3-type plasmid and disseminates among different isolates (Chen et al., 2015). In this study, we analyzed the genomic sequence of the NDM-7-producing ST34 *K. pneumoniae* strain and the genetic surroundings and molecular characterization of NDM-7.

## Materials and Methods

### Patient and Bacterial Strain

Carbapenem-resistant *K. pneumoniae* was isolated from a bile sample collected from a young woman in Hangzhou First People’s Hospital in March 2017. The strain was identified by matrix-assisted laser desorption ionization-time of flight mass spectrometry (MALDI-TOF-MS, Bruker MALDI-TOF Microflex LT/SH, Bruker Diagnostics, Germany) according to the manufacturer’s instructions. The patient had no history of traveling abroad. Informed consent was obtained for this study. The methods in this study were approved by the institutional ethics committee of Hangzhou First People’s Hospital and were carried out in accordance with the approval guidelines.

### Antimicrobial Susceptibility Testing and Detection of Carbapenemase

Antimicrobial susceptibility was determined by the VITEK compact II automated microbiology system and interpreted according to the Clinical and Laboratory Standards Institute guidelines in 2018 (CLSI). The modified carbapenemase inactivation method (mCIM) and EDTA-modified mCIM (eCIM) were used to detect carbapenemase and metallo-β-lactamase as recommended by the CLSI in 2018 (CLSI, 2018).

### Detection of Carbapenemase Genes

PCR was performed to screen carbapenem resistance genes, including *bla*_*KPC*_, *bla*_IMP_, *bla*_*VIM*_, *bla*_NDM_, and *bla*_*OXA–*48_. The amplicons were sequenced by Sanger sequencing.

### Conjugation and Transformation Experiments

To evaluate the horizontal transferability of *bla*_NDM_, mixed broth mating was used as described in a previous study (Du et al., 2017). Sodium azide-resistant *E. coli J53* was used as the recipient strain (donated by Professor YU, Sir Run Run Shaw Hospital, College of Medicine, Zhejiang University). The transconjugants were selected on MacConkey agar containing 100 mg/liter sodium azide and 2 mg/liter meropenem for 24 h at 37°C. The electrotransformation assay was also performed to evaluate the dissemination of *bla*_NDM_ using *DH5*α as the recipient strain as in a previous study (Findlay et al., 2017). The presumptive transconjugants were selected on MH agar plates supplemented with 2 mg/liter meropenem. All successful transformants were confirmed for the presence of *bla*_NDM_ by PCR, and an antimicrobial susceptibility test was performed using the *E*-test method in parallel with the original strains and donor strains.

### Whole Genome Sequencing

The DNA of HZW25 was extracted according to the manufacturer’s instructions and sequenced with Pacific Biosciences RSII SMRT technology SMRT technology (Menlo Park, CA, United States). Sequence reads were assembled using a hierarchical genome assembly process (HGAP) compiled specifically for quality trimming and *de novo* assembly. The graphical maps of the whole genome were converted by BLAST Ring Image Generator (BRIG). The whole-genome sequence was annotated using the Prokaryotic Genomes Automatic Annotation Pipeline (PGAAP) server available at NCBI.^1^ Multilocus sequence typing (MLST) analysis of HZW25 and cgMLST phylogenetic relationship analysis of all public sequences of ST34 were performed using the BacWGSTdb server with the entire genome sequence (Ruan and Feng, 2016). The antibiotic resistance genes were determined using ResFinder 3.0 with > 97% gene identity threshold (exception of β-lactamase variants with 100% identity) and 100% gene length and the comprehensive antibiotic resistance database (CARD) at https://card.mcmaster.ca/ with resistance gene identifier of > 90% identity. The virulence factors were detected using the virulence database of *K. pneumoniae*^2^ and a virulence factor database.^3^

### Plasmid Analysis

All plasmid sequences were annotated using the PGAAP server. The plasmid replicon type was determined by PlasmidFinder^4^ with a 95% threshold for identity and 100% coverage. The *bla*_NDM–7_-carrying plasmid was compared to the publicly available plasmid references using BLAST at GenBank (www.ncbi.nlm.nih.gov/GenBank/). The plasmid comparisons were generated by Easyfig according to the online protocol,^5^ and presentations were generated by EdrawMax. The genetic environment of *bla*_NDM–7_ was analyzed and compared that of *bla*_NDM_ variants.

### Nucleotide Sequence Accession Number

The complete nucleotide sequence of HZW25 and four plasmids were deposited under the GenBank accession numbers CP025211–CP025215.

## Results

### Susceptibility Test

HZW25 was resistant to all β*-*lactams tested, while it was susceptible to aminoglycosides and fluoroquinolones. Carbapenemase activity was positive with mCIM and eCIM tests, suggesting the production of metallo-β-lactamase (MBL).

### Antimicrobial Resistance Genes and Transfer Experiments

The acquired antibiotic resistance genes, including *bla*_NDM–7_, *bla*_SHV–26_, *fosA*, *oqxA*, and *oqxB*, were responsible for the resistance profile of HZW25. In addition, intrinsic antibiotic resistance genes with CARD resistance gene identifiers were also identified, including antibiotic efflux pumps of the major facilitator superfamily (MFS) (KpnE, KpnF, KpnG, KpnH, and ermR), an ATP-binding cassette (ABC) antibiotic efflux pump (msbA), regulators of the efflux pump (marA and marR), the porin membrane protein OmpK35 and a bleomycin resistance gene (BRP). There were no mutations in resistance genes on the chromosome. Only *bla*_NDM–7_ was located on the plasmid, while the other resistance genes were located on the chromosome. *bla*_NDM–7_ was successfully transferred to *E. coli AzR J53* by conjugation and to *E. coli DH5*α by electroporation, and the transconjugants displayed resistance to broad-spectrum cephalosporins and carbapenems (Table 1). The presence of the *bla*_NDM–7_ gene in transconjugants was confirmed by PCR.

**TABLE 1:** The minimum inhibitory concentration to antibiotics of transformed isolates with *E*-test methods (ug/mL).

	CRO	AMC	CTX	FEP	SCF	MEM	IPM	CIP	LEV	MH	ATM	TM	CN
HZW25:J53	>256	>256	>256	>256	>256	>32	>32	0.008	0.023	1	0.064	0.25	0.25
J53	<0.064	4	<0.064	<0.064	<0.064	<0.008	0.5	<0.008	0.023	1	0.064	0.25	0.25
DH5a:pHZW25	>256	>256	>256	>256	256	32	>32	0.008	0.023	0.5	0.064	0.25	0.25
DH5a	<0.064	2	<0.064	<0.064	<0.064	<0.008	0.008	<0.008	0.023	0.5	<0.064	0.25	0.25
HZW25	>256	>256	>256	>256	>256	>32	>32	0.25	0.75	>256	0.5	0.25	0.25

*CRO, Ceftriaxone; AMC, Amoxicillin-clavulanic acid; CTX, Cefotaxime; FEP, Cefepime; SCF, Sulbactam and Cefoperazone; MEM, Meropenem; IPM, Imipenem; CIP, Ciprofloxacin; LEV, Levofloxacin; MH, Minocycline; ATM, Aztreonam; TM, Tobramycin; CN, Gentamicin.*

### Molecular Grouping and Whole Genome Sequencing

**FIGURE 1:**
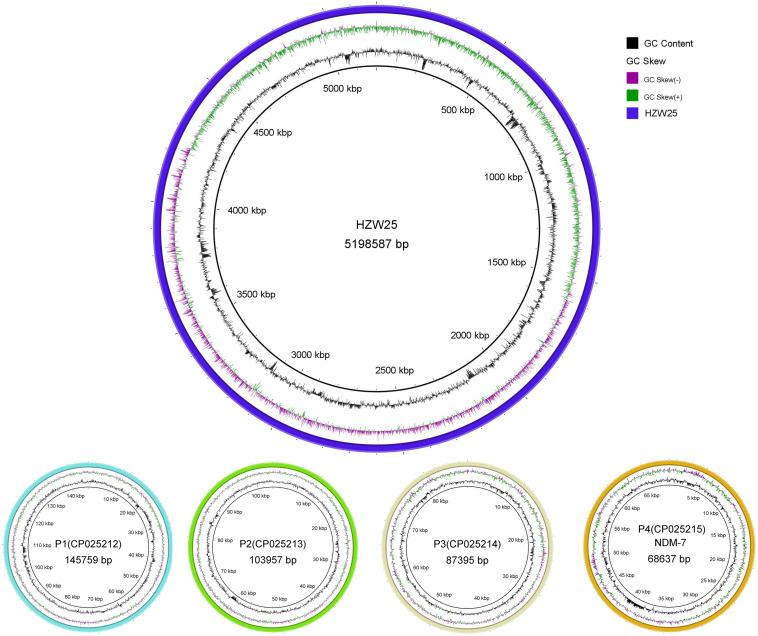
The complete genome of *K. pneumoniae* strain HZW25.

**FIGURE 2:**
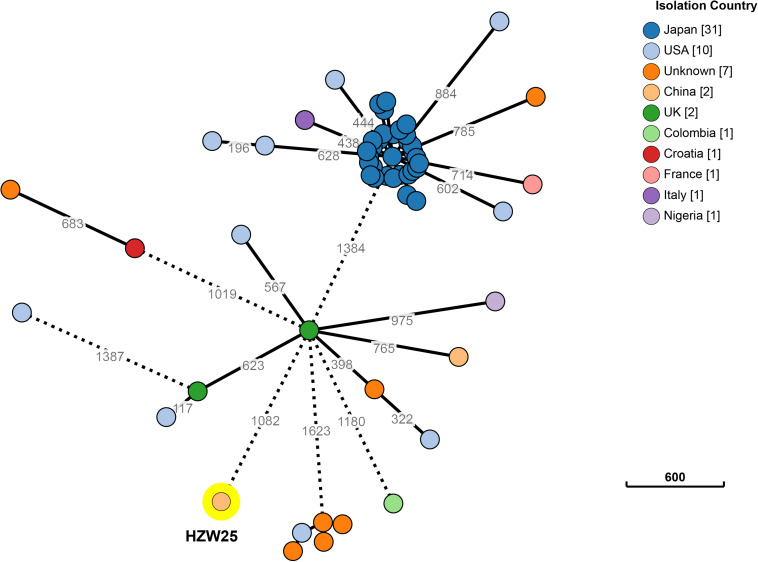
The phylogenetic trees of all ST34 *K. pneumoniae* strains of released public sequences. The evolutionary distance showed that HZW25 was far from the other strains.

Approximately 1.2 Gb clean data was generated after whole genome sequencing with Pacific Biosciences RSII SMRT technology, providing a 221.0-fold average coverage of the genome. The data were assembled into five contigs, which contained one chromosome and four plasmids (Figure 1). HZW25 belonged to ST34 with the MLST sequence 2-3-6-1-9-7-4. The isolate was negative for the string test due to the deficiency of the *rmpA* and *rmpA2* genes. HZW25 belonged to the K73 capsular type as determined by the *wzi* gene encoding the outer membrane protein of the cluster. Virulence genes were present in HZW25, including the *mrkABCDFHIJ* operon encoding type 3 fimbriae for biofilm formation, the *fimABCDEFGHIK* operon encoding type 1 fimbriae for adherence, the *iutA* gene encoding aerobactin, the *entABCDEFS* operon and the *fepABCDG* operon encoding enterobactin siderophore, and the *iroE* and *iroN* genes encoding salmochelin. HZW25 also had a secretion system including the *clpV* gene encoding T6SS-II, *dotU*, the *impAFGHJ* operon, *ompA* and *sciN* encoding T6SS-III. The four plasmids were designated P1, P2, P3 and P4, and they belonged to IncFIB(K), IncR, IncFII(pKPX1), and IncX3, respectively. The plasmids were characterized, and the results are shown in Table 2. Only P4 carried a resistance gene with *bla*_NDM–7_, and the other plasmids harbored no resistance genes. To date, there have been 57 *K. pneumoniae* strains belonging to ST34 worldwide, including Japan (*n* = 31), the United States (*n* = 10), China (*n* = 2), the United Kingdom (*n* = 2) and other countries (*n* = 12). Almost all strains were collected from humans and carried the *bla*_SHV–26_, *fosA*, *oqxA*, and *oqxB* resistance genes, but only HZW25 had *bla*_NDM_ variants of *bla*_NDM–7_. Thirty-one strains from Japan carried *bla*_IMP–1_ and *bla*_CTX–M–2_ in addition to *bla*_SHV–26_ (Supplementary 1). All chromosomes of the ST34 *K. pneumoniae* strains harbored virulence clusters, such as the type I fimbriae cluster *fim* operon, type III fimbriae cluster *mrk* operon, and enterobactin siderophore cluster *fep* and *ent* operon (Supplementary 1). The phylogenetic trees of all ST34 *K. pneumoniae* strains in GenBank revealed that HZW25 was in a separate cluster (Figure 2), suggesting that the HZW25 strain had a long evolutionary distance from the others.

**TABLE 2:** The genomic information of HZW25 and four plasmids.

	Genome length	Number of genes	Plasmid type	Resistant genes	Accession number
HZW25	5 198 587	5 776	–	SHV-26, oqxA, oqxB, fosA	CP025211
P1	145 759	174	IncFIB(K)	–	CP025212
P2	103 957	112	IncR	–	CP025213
P3	87 395	123	IncFII(pKPX1)	–	CP025214
P4	68 637	93	IncX3	NDM-7	CP025215

### Characterization and Genetic Context of the *bla*_NDM–7_ Gene

**FIGURE 3:**
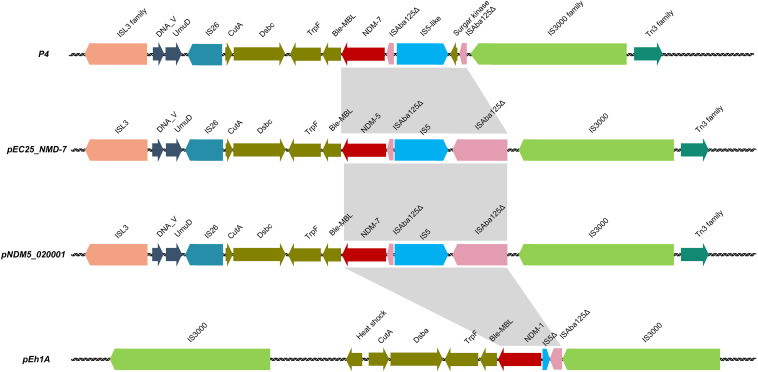
The comparative genetic context of *bla*_NDM_ variants between *P4*, *pEC25_NMD-7, pNDM5_020001*, and *pEH1A.*

The *bla*_NDM–7_ gene was localized in plasmid P4 and could be horizontally transferred successfully from the donor strain HZW25 to the recipient strains *E. coli J53* and *E. coli DH5*α. P4 was 68 637 bp in size with a G + C content of 45.69% and 93 open reading frames. Comparative DNA sequence analysis showed that P4 possessed an IncX3-type backbone. A comparison of the whole region between *P4, pEC25_NMD-7, pNDM5_020001*, and *pEh1A* showed that the genetic context of the regions flanking the NDM variants was similar, and the backbone of plasmids showed high degrees of conversation and similarity. The NDM variant transposable elements of *P4*, *pEC25_NMD-7* and *pNDM5_020001* were highly similar with the *ISL3* family and *IS3000* transposons. Δ*ISAba125* was upstream of the *bla*_NDM_ variants and was inserted by the *IS5* or Δ*IS5* sequence (Figure 3).

## Discussion

Carbapenemase-resistant *K. pneumoniae* (CRKP) has been a significant clinical problem with very limited therapeutic options (Lee et al., 2016). Due to the high-level resistance of CRKP to almost all antibiotics and the high mortality rate of CRKP infections, carbapenemase-producing carbapenem resistance is a public health threat (Lee et al., 2016; Zhang et al., 2018).

In China, CRE strains are mainly composed of KPC-producing *K. pneumoniae* and NDM-producing *E. coli* (Zhang et al., 2017, 2018). NDM-7 has been reported in *E. coli* strains (Hao et al., 2018). Recently, NDM-7 carrying the IncA/C2 type plasmid harbored by *K. pneumoniae* strain ST147 was reported (Shankar et al., 2019). In our study, *bla*_NDM–7_ was first reported in ST34 *K. pneumoniae*. HZW25 was isolated from a young woman with acute cholangitis infection; based on multilocus sequence typing, HZW25 belongs to ST34 and contains several resistance genes responsible for beta-lactam resistance. The *bla*_NDM–7_ gene was located on an IncX3 plasmid, and the genetic environments and characteristics of the *bla*_NDM–7_-producing plasmid P4 were analyzed. The module of NDM-7 transposable elements was highly similar to the *bla*_NDM_ variants harboring the IncX3-type plasmid, suggesting that this NDM variant module could disseminate among different clones.

In recent years, NDM variants, including NDM-7, have been increasingly reported in CRE isolates (Khan et al., 2017; Wu et al., 2019). In contrast to foreign countries, such as India, Spain and Canada, where NDM-7-producing strains are mainly *K. pneumoniae* strains (Chen et al., 2015; Seara et al., 2015; Lynch et al., 2016; Lazaro-Perona et al., 2017), in China, NDM variants are more common in carbapenemase-producing *E. coli* (Bi et al., 2018; Wang et al., 2018; Zhang et al., 2018). Wang L reported NDM-7-producing uropathogenic *E. coli* from a patient with bacteriuria in 2016 (Wang et al., 2016). Bi R reported a high prevalence of *bla*_NDM_ variants in CRE *E. coli* strains, and *bla*_NDM–7_ ranked third among those variants (Bi et al., 2018). Hao Y analyzed the genotypic and phenotypic characterization of *bla*_NDM–7_ in *E. coli* from a patient with a urinary tract infection (Hao et al., 2018). Recently, Xu J and He F also reported NDM-7-producing *E. coli* from a urinary sample (Xu and He, 2019).

Although *bla*_NDM_ variants are both located on bacterial chromosomes and plasmids, *bla*_NDM_ variants positioned on plasmids play a vital role in the dissemination of resistance genes (Bonnin et al., 2012; Ho et al., 2012). *bla*_NDM_ variants harboring plasmids are mainly of the IncFII, IncX3, and IncC(IncA/C2) types (Perez-Vazquez et al., 2019). IncX3 is the most common type of plasmid carrying *bla*_NDM_, and most IncX3 plasmids are present in *E. coli* (Paul et al., 2017; Bi et al., 2018). From the worldwide distribution of *bla*_NDM_-carrying plasmids in *Enterobacteriaceae*, IncX3 plasmids may serve as an important vehicle in the dissemination of NDM in East Asia, particularly in China (Ho et al., 2012). Hao Y compared the backbones of plasmids carrying NDM variants and collected from human and food animal origin, and the results showed that all plasmids were highly similar (> 99%) among patients (Hao et al., 2018). This suggested that IncX3 plasmids may serve as one of the major platforms on which *bla*_NDM_ genes evolve with the generation of new NDM variants. Lee CS isolated NDM-7-producing *K. pneumoniae* and *E. coli* simultaneously from a patient, suggesting that *bla*_NDM–7_ might be transferred between *K. pneumoniae* and *E. coli in vivo* (Lee et al., 2014).

In our study, *bla*_NDM–7_ was in a 68 637 bp IncX3-type plasmid. *bla*_NDM–7_ was successfully transferred to *E. coli AzR J53* by conjugation and to *E. coli DH5a* by electroporation. Compared to the common genetic contexts of *bla*_NDM_ variants, *bla*_NDM–7_ had a similar surrounding environment, with *ISAba125* (intact or truncated) upstream and *ble*_*MBL*_ downstream; these elements are located in the transposon-like structure flanked by *IS3000* and *IS26*, responsible for horizontal transfer of *bla*_NDM–7_ among *Enterobacteriaceae*.

The *bla*_NDM–7_ gene can be carried by different *K. pneumoniae* strains of different STs, including ST147, ST138, ST273, ST437, and ST278 (Lee et al., 2014; Chen et al., 2015; Seara et al., 2015; Chou et al., 2016; Lynch et al., 2016; Perez-Vazquez et al., 2019; Shankar et al., 2019). Although these strains were isolated in different countries and regions, these strains contain plasmids carrying *bla*_NDM–7_ with similar surroundings and are characterized by horizontal gene transmission (Chen et al., 2015). Chen described *bla*_NDM_-carrying plasmids from different *Enterobacteriaceae* isolates with identical structures, suggesting that very effective horizontal transfer events had occurred (Chen et al., 2015). Seara N reported the interhospital spread of NDM-7-producing *K. pneumoniae* in Spain (Seara et al., 2015). In our study, ST34 *K. pneumoniae* carrying *bla*_NDM–7_ was reported for the first time, and the evolutionary distance from known ST34 *K. pneumoniae* strains showed that the strain we isolated was far from the other isolated strains.

Ceftazidime-avibactam is an effective antibiotic for CRE. It has good antibacterial activity against KPC-producing *K. pneumoniae* but not NDM-producing *K. pneumoniae* or *E. coli* (van Duin and Bonomo, 2016; Shirley, 2018). The emergence of NDM-producing *K. pneumoniae* would be troublesome in CRE treatment using ceftazidime-avibactam. Therefore, it is important to screen carbapenemase types before ceftazidime-avibactam therapy, and it is necessary to detect serine proteases and metalloproteinases in epidemiological investigations.

## Conclusion

This study describes *bla*_NDM–7_ in an ST34 *K. pneumoniae* strain for the first time. The *bla*_NDM–7_ gene is located on a conjugated and horizontally transmitted IncX3-type plasmid. The potential dissemination of *bla*_NDM_-like genes in IncX3-type plasmids should be considered. Before treatment with ceftazidime-avibactam, it is necessary to determine the types of carbapenemase in carbapenemase-resistant *K. pneumoniae.*

## Data Availability Statement

All datasets presented in this study are included in the article/Supplementary Material.

## Author Contributions

MW, XW, and DY provided assistance and guidance in the research. QC and JZ wrote the manuscript. SW and YY assisted the manuscript checking. All authors checked the manuscript and submitted the final version.

## Conflict of Interest

The authors declare that the research was conducted in the absence of any commercial or financial relationships that could be construed as a potential conflict of interest.

## Footnotes

1 http://www.ncbi.nlm.nih.gov/

2 https://bigsdb.pasteur.fr/cgi-bin/bigsdb/bigsdb.pl?db=pubmlst_klebsiella_seqdef&page=sequenceQuery

3 http://www.mgc.ac.cn/VFs/

4 https://cge.cbs.dtu.dk/services/PlasmidFinder/

5 https://mjsull.github.io/Easyfig/
